# Myelin Repair: From Animal Models to Humans

**DOI:** 10.3389/fncel.2021.604865

**Published:** 2021-04-14

**Authors:** Myriam Cayre, Marie Falque, Océane Mercier, Karine Magalon, Pascale Durbec

**Affiliations:** Aix Marseille Université, Centre National de la Recherche Scientifique (CNRS), Institut de Biologie du Développement de Marseille (IBDM-UMR 7288), Marseille, France

**Keywords:** myelin repair, oligodendrocyte, neural stem cells, subventricular zone, multiple sclerosis, therapeutic strategies

## Abstract

It is widely thought that brain repair does not occur, but myelin regeneration provides clear evidence to the contrary. Spontaneous remyelination may occur after injury or in multiple sclerosis (MS). However, the efficiency of remyelination varies considerably between MS patients and between the lesions of each patient. Myelin repair is essential for optimal functional recovery, so a profound understanding of the cells and mechanisms involved in this process is required for the development of new therapeutic strategies. In this review, we describe how animal models and modern cell tracing and imaging methods have helped to identify the cell types involved in myelin regeneration. In addition to the oligodendrocyte progenitor cells identified in the 1990s as the principal source of remyelinating cells in the central nervous system (CNS), other cell populations, including subventricular zone-derived neural progenitors, Schwann cells, and even spared mature oligodendrocytes, have more recently emerged as potential contributors to CNS remyelination. We will also highlight the conditions known to limit endogenous repair, such as aging, chronic inflammation, and the production of extracellular matrix proteins, and the role of astrocytes and microglia in these processes. Finally, we will present the discrepancies between observations in humans and in rodents, discussing the relationship of findings in experimental models to myelin repair in humans. These considerations are particularly important from a therapeutic standpoint.

## Introduction

Myelin is an insulating sheath that surrounds the axons. It allows the propagation of saltatory impulses, resulting in an efficient acceleration of signal conduction along the axons, together with protection and metabolic support for neurons.

Many studies have suggested that there is a correlation between myelin content and cognitive function. According to the recently developed concept of “myelin plasticity,” experience-dependent changes in myelin may modulate brain function (Purger et al., 2016) through effects on signal synchronization (Bells et al., 2019; Hasan et al., 2019).

Within the central nervous system (CNS), myelin is produced by oligodendrocytes (OLGs). These cells produce huge amounts of cytoplasmic membrane, which is then wrapped around the axons. The myelin membrane is unique in that 70% of its dry weight consists of lipids, including cholesterol and galactolipids in particular, and it contains a specific set of proteins including proteolipid protein (PLP) and myelin basic protein (MBP). The membrane loops are compacted to form myelin segments. During embryonic development, oligodendrocyte progenitor cells (OPCs) are specified from neuroepithelial neural stem cells in the spinal cord and the forebrain (Rowitch and Kriegstein, 2010). Before birth, the OPCs proliferate and migrate throughout the CNS parenchyma, but myelination occurs principally during the postnatal period, when OPCs differentiate and become myelinating OLGs. The first OLGs expressing myelin proteins appear as early as mid-gestation in humans, but brain myelination is not complete until several weeks after birth in rodents and several years after birth in humans. Myelin is then continually remodeled throughout life, through several different processes: the addition of myelin segments, lengthening, retraction, and changes in thickness. Interestingly, a fraction of OPCs in the adult brain remain at the stage of immature progenitors and can respond to neuronal activity by proliferation and differentiation (Gibson et al., 2014), to adapt myelination to the needs of active networks. This adaptive myelination is required for the correct learning of new complex tasks, as shown by the impaired motor learning and fear memory of healthy adult mice in which the differentiation of OPCs is prevented (McKenzie et al., 2014; Xiao et al., 2016; Pan et al., 2020). Myelin is now considered to play a key role in the correct functioning of the brain.

Unfortunately, myelin may be injured or degraded and is affected by aging. OLGs produce large amounts of cell membrane, and their metabolic rates are, therefore, high. OLGs also have low reduced glutathione levels and a high iron content, rendering them susceptible to oxidative stress, excitotoxic damage, and inflammation (Juurlink et al., 1998; Benarroch, 2009). Deficiencies of glycolysis in OLGs were recently shown to trigger inflammasome activation, ultimately leading to demyelinaton (Zhang et al., 2020).

Consequently, many traumas, lesions, and infections trigger the death of OLGs, leading to demyelination, which contributes to functional disorders. Demyelination induces axonal defects, such as nodal complex alterations, conduction blocks, and/or irreversible axon losses that clearly disrupt long-range connectivity (Nave, 2010), as in multiple sclerosis (MS), an autoimmune disease targeting OLGs. Together, these disturbances cause motor, sensory, and cognitive dysfunctions.

## Remyelination is a major Issue for Preventing Neurodegeneration and Irreversible Losses of Function

OLGs and axons are intricately connected, and this relationship is crucial for the preservation of axon integrity and correct signal conduction. The preservation of this “neuron–OLG” unit not only guarantees the maintenance of saltatory conduction, but also provides the neurons with neuroprotective trophic support. Indeed, the myelin sheath plays an important role in ensuring axon survival, by providing physical and metabolic support (Fünfschilling et al., 2012; Lee et al., 2012), and OLG dysfunction is sufficient to trigger neuronal death (Montag et al., 1994; Griffiths et al., 1998; Lappe-Siefke et al., 2003). In pathological conditions, denuded axons are exposed to a toxic inflammatory environment, in which early remyelination may limit excessive neuronal death by insulating the axons against the potentially hostile microenvironment. Several studies of the transplantation of myelin-forming cells have provided strong support for the concept that timely remyelination protects axons from degeneration and promotes functional recovery, in both MS (Irvine and Blakemore, 2008) and spinal cord injury models (Karimi-Abdolrezaee et al., 2006; Hawryluk et al., 2014; Nagoshi et al., 2018). Accelerating remyelination in experimental autoimmune encephalitis (EAE), a model of inflammatory demyelination, is also sufficient to preserve axonal integrity and neuronal function (Mei et al., 2016). During demyelination, the nodes of Ranvier are disorganized, altering nerve conduction. Following remyelination, the nodes are reorganized, allowing functional restoration, within a critical time window, consistent with the notion that node restoration is more beneficial if initiated before the occurrence of axonal damage (Saifetiarova et al., 2018). Nevertheless, the remyelination of damaged axons remains possible and may promote axonal recovery and, thus, neuronal survival (Schultz et al., 2017). Finally, remyelination index, which varies considerably between MS patients, leading to the classification of patients as “good” and “bad remyelinators,” is highly correlated with functional outcome (Bodini et al., 2016).

Most neurons do not regenerate (with the exception of neurogenic niches in the dentate gyrus and subventricular zone), but spontaneous myelin repair is commonly observed. This process is highly efficient in rodent models, in which complete remyelination occurs within weeks or months following the experimental insult; by contrast, remyelination varies considerably between MS patients and between individual lesions in the same patient (Patrikios et al., 2006; Albert et al., 2007; Patani et al., 2007; Bodini et al., 2016). It is, therefore, crucial to understand all the elements at work in this regenerative process fully, to facilitate the identification of new targets and the development of therapeutic strategies.

## Cell Sources for Myelin Repair: What We Have Learned From Animal Models

The complex nature of MS makes it difficult to mimic the disease faithfully in animal models, but these models have nevertheless been the source of most of our knowledge on the cell biology of myelin repair. The most widely used models of demyelination are as follows (Burrows et al., 2019): (1) focal demyelination of the white matter by the intracerebral injection of demyelinating agents, such as lysophosphatidylcholine (LPC, a membrane-dissolving agent) or ethidium bromide (a DNA-intercalating agent), which trigger rapid demyelination (over a period of a few hours for LPC, to a few days for ethidium bromide), followed by complete remyelination over a period of 2–3 weeks; (2) massive and widespread demyelination of the brain by the ingestion of cuprizone (a copper chelator) in feed, followed by complete remyelination in the acute (3–6 weeks of treatment) model, or incomplete remyelination in the chronic model (12 weeks of treatment); and (3) EAE, in which inflammation is the predominant feature observed after immunization. EAE can be triggered by two approaches: active immunization with myelin peptides or passive induction *via* the adoptive transfer of activated myelin-specific Th1 or Th17 cells from immunized donors in naïve syngeneic recipients. EAE has proved very useful for studies of the pathogenesis of the disease and the role of immune cells, but the demyelination lesions generated are highly variable in size and unpredictable and occur at different stages of development. Furthermore, axonal integrity is compromised in this model, making it difficult to study remyelination. For these reasons, the LPC and cuprizone models (in which extensive demyelination is followed by robust remyelination) are preferred for studies of the cellular mechanisms of demyelination and remyelination. However, these models do not encompass the complexity of MS pathogenesis due to the absence of adaptive immune system involvement.

**Figure 1:**
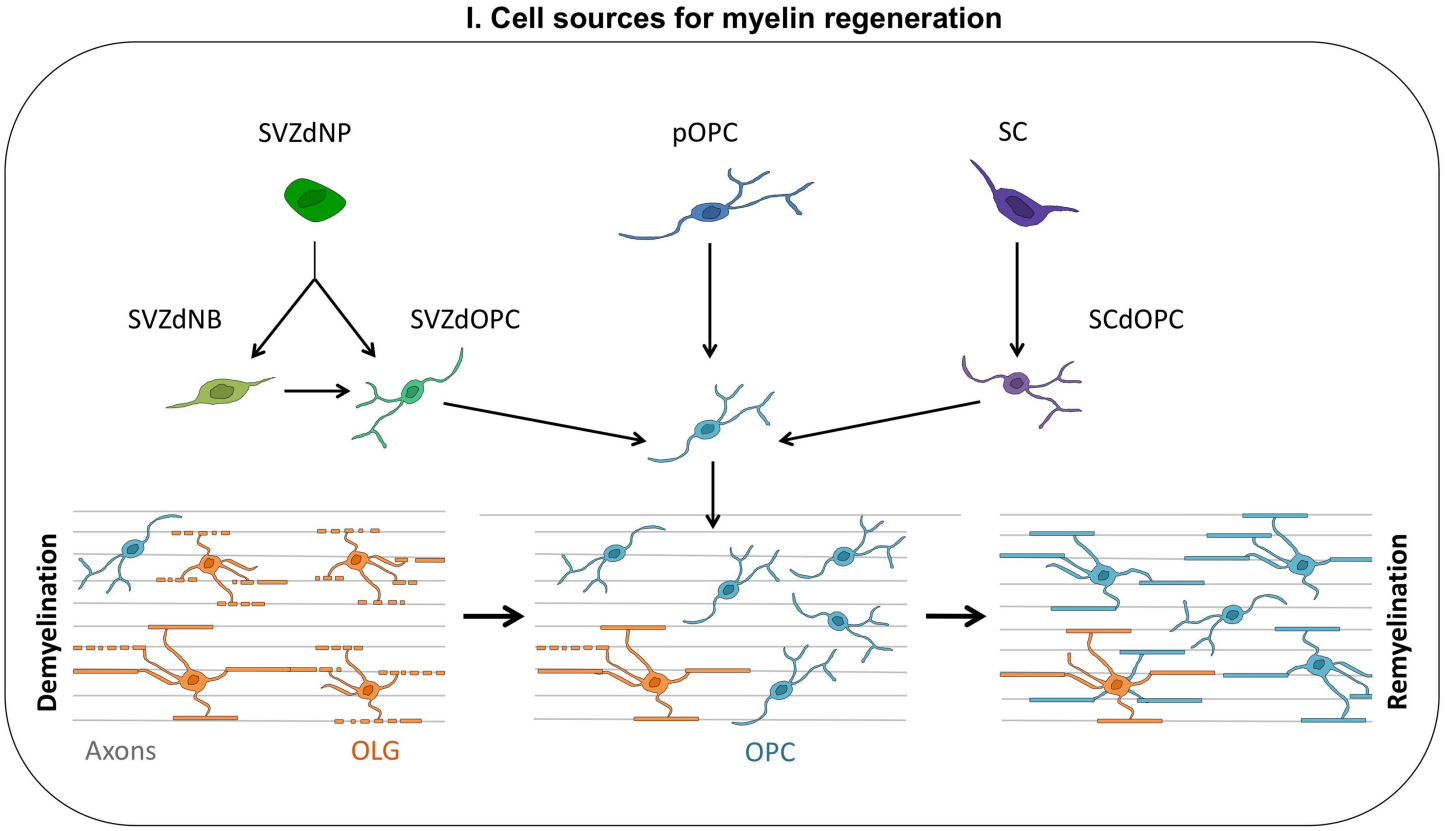
Cell sources for myelin regeneration. Following a demyelination insult, oligodendrocyte progenitor cells (OPCs) are mobilized: they proliferate, migrate toward the injury, and finally differentiate into new myelinating oligodendrocytes (OLGs). These OPCs contributing to myelin repair may be derived from the subventricular zone (SVZdOPC) [directly or by reprogramming of neuroblasts (SVZdNB)], from embryo-derived OPCs (pOPC) or from Schwann cells (SCdOPC). Surviving OLG can also produce new myelin segments thus contributing to remyelination.

Progress in mouse genetic techniques opened up opportunities for lineage cell tracing, making it possible to identify the different cell types contributing to OLG replacement and remyelination (Figure 1). The data obtained in rodents concerning the cells involved in myelin regeneration are summarized below.

### Oligodendrocyte Progenitor Cells

Most of the OPCs present in adult rodent brain are generated from the neuroepithelium during embryonic development; then they proliferate and disseminate throughout the brain parenchyma. OPCs are often characterized by NG2 and PDGFRa expression. They constitute the major population of dividing cells in the healthy adult brain. In physiological conditions, they have a very long cell cycle, with a prolonged G1 phase, and only a minority of these cells differentiate into OLGs (Dimou et al., 2008).

#### OPC Reactivity Following Demyelination

Early studies reported a rapid increase in OPC density following demyelination (Franklin et al., 1997; Levine and Reynolds, 1999; Reynolds et al., 2001; Chari and Blakemore, 2002), due to rapid recruitment of the pool of NG2-expressing cells through a shortening of their cell cycle (Simon et al., 2011) and the stimulation of short-distance migration toward the site of the lesion (Franklin and Blakemore, 1997). Indeed, in demyelinating conditions, OPCs are activated and revert to a more juvenile phenotype; they then produce the cytokine IL-1β and the chemokine CCL2, which enhance OPC mobilization and the repopulation of demyelinated areas (Moyon et al., 2015). OPC density gradually returns to normal levels with the reappearance of mature OLGs in the lesion. These observations suggested that the parenchymal OPC pool was responsible for spontaneous remyelination. This was definitively demonstrated by lineage cell tracing with PDGFRaCreERT2:RosaYFP and NG2CreERT2:TaumGFP mice, in which OPCs and their progeny were labeled following tamoxifen injection. These studies provided direct evidence for the generation of remyelinating oligodendrocytes from OPCs (Zawadzka et al., 2010; Mei et al., 2016).

#### OPC Diversity and Remyelination

There is growing evidence to suggest that OPCs do not constitute a homogeneous cell population (for a review, see Werkman et al., 2020). However, it is difficult to differentiate between real cell diversity and different lineage stages within the same cell population, because only a few studies have shown phenotypic differences to be intrinsic and associated with functional specificity (Foerster et al., 2019).

The first evidence for OPC heterogeneity was provided by the difference in cell cycle kinetics between OPCs residing in the white and gray matter. The OPC cell cycle in the corpus callosum (CC) of young adult mice takes about 7 days, whereas that in the cortex takes between 21 and 50 days (Dimou et al., 2008; Rivers et al., 2008; Simon et al., 2011; Young et al., 2013). The OPCs in the white and gray matter also differ in terms of their differentiation potential. About 40% of the OPCs in the CC differentiate into OLGs, vs. only 11% in the cortex (Dimou et al., 2008). Grafting experiments have suggested that these differences are due to both the intrinsic properties of the OPCs and environmental cues (Viganò et al., 2013). Cultured rat gray matter OPCs have a less mature phenotype (in terms of morphology and gene expression), higher rates of proliferation, and slower differentiation than white matter OPCs. Furthermore, migration in response to cues secreted by astrocytes is weaker in white matter OPCs, which are more sensitive to the inhibition of proliferation and differentiation by TNFα and IFNγ (Lentferink et al., 2018). In the context of demyelination, these characteristics may confer advantages on advantages to gray matter OPCs.

The heterogeneity of OPCs is also revealed by their electrophysiological properties. OPCs are sensitive to neuronal activity due to the expression of ion channels and neurotransmitter receptors. However, not all OPCs are excitable, and white and gray matter OPCs have different electrophysiological signatures, often linked to their differentiation potential (Spitzer et al., 2019).

Recent studies have revealed not all OPCs contribute equally to myelin repair. During embryonic development, OPCs arise from different neuroepithelium domains. In response to demyelination in the adult brain, OPCs from dorsal origin are more strongly mobilized than ventral OPCs, but these cells are more sensitive to the age-related decline in differentiation potential (Crawford et al., 2016b). This is one of the rare examples of the functional diversity of OPCs. A subset of OPCs evenly distributed throughout the brain and characterized by the expression of the G protein-coupled receptor GPR17 has been identified as a reserve pool for repair purposes (Lecca et al., 2008; Viganò et al., 2016). In physiological conditions, GPR17 is required to initiate OPC differentiation, but must be downregulated to allow cells to undergo terminal maturation. Thus, in the absence of lesions, the subpopulation of GPR17^+^ OPCs is quiescent progenitors that do not differentiate into OLGs (Fumagalli et al., 2017). GPR17 is activated by purines and leukotrienes, the levels of which increase after lesion formation. In MS models, a robust induction of GRP17 is observed in OPCs, together with a large increase in OPC proliferation in the CC (Coppolino et al., 2018; Nyamoya et al., 2019). However, although these activated progenitors produce mature OLGs in the cuprizone model, the process fails in EAE, suggesting that inflammation (in EAE) may lead to an overactivation of GPR17, thereby preventing the terminal differentiation of OPCs (Coppolino et al., 2018). GPR17 reactivity is not observed in the cortex of cuprizone-fed mice (Nyamoya et al., 2019). These results suggest that GPR17 may be a suitable molecular target for the promotion of endogenous myelin repair.

Another subpopulation of OPCs has been identified on the basis of levels of expression for ITPR2, an intracellular calcium channel. Motor learning strongly stimulates the production of ITPR2^+^ OPCs, which then differentiate into OLGs, contributing to early learning by facilitating electrical transmission (Marques et al., 2016).

These differences probably account for remyelination occurring later in the cortex than in the CC: after acute cuprizone-induced demyelination, OPC repopulation and differentiation is much faster in the CC than in the cortex, resulting in later, incomplete remyelination in the cortex (Gudi et al., 2009; Baxi et al., 2017; Nyamoya et al., 2019). However, in a chronic model based on cuprizone treatment associated with rapamycin to inhibit OPC differentiation so as to improve reproduction of the characteristics of chronic MS, remyelination was faster and more robust in the cortex than in the CC (Bai et al., 2016), as observed in patients with chronic MS (Chang et al., 2012). In both cases (cuprizone model and patients), these differences in remyelination efficiency were associated with differential astrocyte reactivity in the cortex and CC (see below for the role of astrocytes in myelin repair).

### Subventricular Zone-Derived Neural Progenitors

#### Evidence for Subventricular Zone-Derived Oligodendrogenesis

In the adult brain, the wall of the lateral ventricle is a stem cell niche with a unique cellular and extracellular organization (Mirzadeh et al., 2008; Tavazoie et al., 2008; Mercier, 2016). The neural stem cells present in the subventricular zone (SVZ) persist throughout life; they are quiescent but can be activated and re-enter the cell cycle when needed. They give rise to amplifying progenitors (“type C” cells), which, in turn, mostly produce neuroblasts that migrate along the rostral migratory stream to the olfactory bulb, where they differentiate into olfactory interneurons (Lois and Alvarez-BuyIIa, 1994; Doetsch et al., 1999). In parallel, a small fraction of C cells give rise to OPCs that invade periventricular areas (CC, striatum) and the cortex (Suzuki and Goldman, 2003; Menn et al., 2006). These low levels of oligodendrogenesis increase by a factor of four after LPC-induced demyelination (Menn et al., 2006), suggesting a role for SVZ-derived OPCs in myelin repair. Substantial levels of progenitor cell emigration from the SVZ to demyelinated lesion sites are also consistently observed in MS models (Nait-Oumesmar et al., 1999; Picard-Riera et al., 2002; Magalon et al., 2007; Cayre et al., 2013). The migration of these cells from their niche to the lesion site is regulated by guidance cues, such as Slit1 (Deboux et al., 2020). Here again, genetic lineage tracing appears to be a useful tool for unequivocally demonstrating the contribution of SVZ-derived progenitors to myelin repair, particularly in periventricular areas, such as the rostrolateral CC (Xing et al., 2014; Brousse et al., 2015). It has also been suggested that SVZ progenitors are an important source of cells for repopulating the parenchymal OPC pool following demyelination-induced pOPC differentiation into myelinating OLGs (Serwanski et al., 2018).

Despite these demonstrations, the role of SVZ-derived progenitors in myelin repair has been called into question in a few studies. Guglielmetti et al. demonstrated, with MRI and bioluminescence imaging, an increase in olfactory bulb neurogenesis in cuprizone-fed mice, but they were unable to detect any migration of SVZ-derived progenitors to the demyelinated CC (Guglielmetti et al., 2014). Another study reported that SVZ progenitors responded rapidly to focal demyelination but failed to produce myelinating OLGs in the lesion (Kazanis et al., 2017). Given the multiple observations based on the genetic tracing and/or immunofluorescence studies of SVZ-derived progenitor cell migration to sites of injury and the differentiation of these cells into OLGs (Xing et al., 2014; Brousse et al., 2015; Samanta et al., 2015; Serwanski et al., 2018; Butti et al., 2019), it seems likely that these noninvasive imaging techniques do not detect mature cells efficiently and lack sensitivity for visualizing the migration of isolated cells.

#### Characterization of the SVZ Progenitors Mobilized

The stem cells in the SVZ are not homogeneous. They are organized into domains according to the combination of transcription factors that confer them functional specificities (Merkle et al., 2014; Bonaguidi et al., 2016). Thus, particular subpopulations of SVZ progenitors may be mobilized after demyelination. A subset of progenitors expressing GLI1, a Sonic Hedgehog effector, has been shown to be particularly responsive after the formation of lesions; surprisingly, the mobilization of these cells is further increased by GLI1 inhibition (Samanta et al., 2015).

Tracing experiments with NestinCreERT2 mice cannot determine the type of progenitors mobilized after demyelination. Neuroblasts have been reported to exit the SVZ and rostral migratory stream following experimentally induced demyelination (Picard-Riera et al., 2002; Cayre et al., 2013), suggesting a possible role in the repair process. Indeed, grafting experiments showed that neuroblasts transplanted into the CC of shiverer mice lacking MBP massively differentiate into myelinating OLGs (Cayre et al., 2006). The upregulation of Chordin in the SVZ of demyelinated mice is partly responsible for this change in fate from neuroblast to OLG (Jablonska et al., 2010). This lineage plasticity was further documented in recent studies showing spontaneous neuroblast conversion into OLGs in cuprizone-fed mice (El Waly et al., 2018). These studies demonstrate that both SVZ-derived OPCs and SVZ-derived neuroblasts contribute to OLG replacement after demyelinating insults. The respective roles of each of these cell populations in the contribution of the SVZ to myelin repair remain unknown.

Bioinformatics and *in silico* genomic analyses have identified a catalog of small molecules as potential regulators of SVZ microdomain-specific lineages (Azim et al., 2017). For example, GSK3β and PI3K/Akt inhibitors have been shown to promote oligodendrogenesis from SVZ neural stem cells and to promote remyelination in response to focal demyelination (Azim et al., 2017). This study provides proof of concept that the pharmacological stimulation of SVZ neural stem cells to produce new OLGs is a potentially valuable strategy for myelin repair.

#### Alternative Roles of SVZ-Derived Progenitors in Myelin Repair

Martino's laboratory investigated the role of SVZ-derived progenitors in myelin repair, using nestin-thymidine kinase-transgenic mice to kill neural progenitors in a specific manner in cuprizone-fed mice (Butti et al., 2019). They concluded that SVZ-derived progenitors were dispensable for remyelination but provided partial protection against greater axonal loss (Butti et al., 2019). Along the same lines, our laboratory showed that some SVZ-derived progenitors mobilized to the demyelinated CC remain undifferentiated and produce factors capable of modulating microglial activation, thereby playing a protective role (Brousse et al., 2020, https://doi.org/10.1101/2020.06.18.158782).

Thus, endogenous SVZ progenitors may promote myelin repair *via* two different mechanisms: OLG replacement and immunomodulation/neuroprotection.

### Schwann Cells

Schwann cells are responsible for peripheral nervous system (PNS) myelination. They are derived from the neural crest during embryonic development. Schwann cells are remarkably plastic and respond to lesions by dedifferentiation and re-entry into the cell cycle, facilitating rapid PNS myelin repair. Early observations described an invasion of the CNS by Schwann cells following LPC-induced demyelination of the spinal cord (Blakemore et al., 1977) and in myelin-deficient mutants (Duncan and Hoffman, 1997). It was originally assumed that Schwann cells invaded the CNS following a breach of the glia limitans, but another mode of Schwann cell contribution to CNS remyelination is now recognized (Blakemore, 2005). Indeed, fate mapping strategies demonstrated that OPCs could differentiate into Schwann cells following CNS demyelination (Zawadzka et al., 2010). After spinal cord injury, extensive Schwann cell-mediated remyelination occurs, much of which is driven by OPC-derived Schwann cells (Assinck et al., 2017). The bone morphogenic protein (BMP)/Wnt pathway partly drives decisions concerning the fate of adult OPCs (differentiation into OLGs or Schwann cells): BMP4 upregulation during demyelination drives OPCs to differentiate into Schwann cells, whereas reactive astrocytes within nonvascular areas inhibit BMP/Wnt, thereby favoring OLG differentiation (Ulanska-Poutanen et al., 2018).

Interestingly, recent studies have shown that the specific deletion of Fbxw7 (a E3 ubiquitin ligase component) in Schwann cells is sufficient to induce the production of thicker myelin sheaths by these cells, together with a myelination of multiple axons similar to that observed with OLGs (Harty et al., 2019). These recent findings raise questions about the relationship between PNS and CNS remyelination and suggest that the demarcation between the biology of Schwann cells and that of OLGs may be less marked than previously appreciated. However, it remains to be demonstrated that Schwann cells can provide appropriate metabolic support to CNS axons and restore effective signal conduction (Chen et al., 2021).

### Mature Oligodendrocytes

Following demyelination, some mature oligodendrocytes are spared and survive. A possible role for these cells in myelin repair was considered but quickly ruled out when it was shown that they do not re-enter the cell cycle and proliferate after experimentally induced demyelination and that they do not migrate to the lesion or extend processes to the lesion site (Keirstead and Blakemore, 1997, 1999; Crawford et al., 2016a). OLGs were, thus, considered to be highly differentiated and specialized cells with a complex morphology and no postlesional plasticity. However, recent studies have suggested that these spared OLGs are not passive witnesses of demyelination and can instead play a significant role in the repair process. OLGs located at the border of the lesion produce heparan sulfates, which, in turn, enhance remyelination *via* Sonic Hedgehog signaling (Macchi et al., 2020). Using 3D electron microscopy in large-animal models (cats and nonhuman primates), Duncan and coworkers provided the first evidence of direct remyelination by mature OLGs (Duncan et al., 2018). The development of *in vivo* two-photon video microscopy with longitudinal follow-up over several weeks subsequently made it possible to demonstrate unequivocally that surviving OLGs were able to generate new myelin sheaths after cuprizone-induced demyelination (Bacmeister et al., 2020). However, the laboratory of Bergles failed to detect such OLG plasticity with similar techniques (Orthmann-Murphy et al., 2020), suggesting that it probably makes only a minor contribution to myelin repair, in particular conditions, such as during motor learning tasks.

In conclusion, four main sources of OLG-forming cells may contribute to remyelination following demyelination: OPCs, SVZ-derived progenitors, Schwann cells, and surviving mature OLGs. OPCs, and OLGs are present throughout the central nervous system and can therefore participate in myelin repair at any lesion site. In this respect, they occupy a privileged position. SVZ-derived progenitors have a strong migratory potential and are equipped to respond to inflammatory cues, but their contribution to myelin repair is unlikely to extend very far from periventricular structures. Remyelination quality may also depend on the source of remyelinating cells. For instance, the myelin sheaths formed by SVZ-derived OLGs appear to be thicker than those formed by OLGs generated from parenchymal OPCs (Xing et al., 2014). Interestingly, surviving mature OLGs produce fewer new myelin sheaths than newly formed OLGs, but they better preserve the pattern of myelination in the cortex, with potential implications for functional recovery (Bacmeister et al., 2020). Indeed, the optimal processing capabilities of cortical circuits may be dependent on specific and stable cortical neuron myelination. Finally, remyelination by Schwann cells leads to myelin sheaths of a different molecular composition, which are not affected in MS patients. This is an interesting and potentially advantageous feature. Conversely, peripheral myelin is less compacted, which may affect conduction efficiency in the CNS. Furthermore, given the 1:1 ratio between Schwann cells and myelin segments, extensive CNS remyelination would require an extremely large number of Schwann cells and little is currently known about the mechanisms driving the differentiation of OPCs into Schwann cells.

## What Curbs Spontaneous Myelin Repair?

Successful remyelination implies progenitor cell proliferation, migration to the lesion site, and differentiation into OLGs. The newly formed OLGs must then engage in dialog with axons, which they must ensheath to form compacted functional myelin sheaths. A glitch in any one of these steps may lead to remyelination failure. We will now consider the conditions that have been shown to inhibit spontaneous myelin repair.

### Aging

Like postlesional regeneration and most plasticity events, the potential for remyelination declines with age. Remyelination is still observed in old rodents, but it is much slower than in younger animals (Shields et al., 1999; Sim et al., 2002). Interestingly, in MS patients, the transition from relapsing–remitting to progressive MS occurs at about the same age, regardless of age at disease onset (Confavreux and Vukusic, 2006b; Tutuncu et al., 2013), suggesting that remyelination failure and disease progression are tightly linked to aging. Aging affects OPC recruitment and differentiation in a cell-autonomous or non-cell-autonomous fashion *via* the alteration of other cell types.

OPCs from aged mice transplanted into neonatal brain recover the proliferation and differentiation rates of newborn OPCs (Segel et al., 2019), suggesting that environmental cues play a crucial role in the age-related decrease in myelin formation. The mechanical properties of the microenvironment may be involved: a recent study revealed that tissue stiffness increases with age, impairing OPC proliferation and differentiation *via* the mechanoresponsive ion channel Piezzo1 (Segel et al., 2019).

The expression profile of growth factors involved in OPC recruitment (PDGFRa, FGF2) and in OPC differentiation (IGF1, TGFβ1) following demyelination is altered in old mice, in which the upregulation of these factors is both weaker and delayed (Hinks and Franklin, 2000).

Consistent with cell-autonomous effects, the epigenetic control of OPC differentiation into myelinating OLGs is disrupted with aging (Shen et al., 2008), and the OPCs of aged mice fail to respond to growth factors and differentiation signals (Neumann et al., 2019). Furthermore, single-cell RNA sequencing-based comparisons of OPCs obtained from young and old mice have revealed mitochondrial dysfunction and a greater activity of the inflammasome and pathways associated with nutrient signaling in aged OPCs (Neumann et al., 2019). These findings were recently confirmed by a proteomic analysis revealing that the levels of proteins associated with oxidative phosphorylation and inflammatory responses increase with age, whereas those of proteins associated with cholesterol biosynthesis and the cell cycle decrease (de la Fuente et al., 2020). Several studies have suggested that, unlike neurogenesis, SVZ-derived oligodendrogenesis does not decline with age (Capilla-Gonzalez et al., 2013; Weissleder et al., 2016). The contribution of SVZ progenitors to myelin repair can also be enhanced by stimulation of the EGF pathway (Aguirre and Gallo, 2007; Cantarella et al., 2008) and enrichment of the environment (Magalon et al., 2007). The SVZ may therefore be an interesting target reservoir for treatments designed to promote myelin repair in elderly patients.

Finally, aging also affects microglial cells and macrophages, which play a crucial role in remyelination, by removing myelin debris that inhibits remyelination (for a review, see Pinto and Fernandes, 2020). Aged microglial cells become dystrophic, and their processes become less motile (Wong, 2013; Hefendehl et al., 2014; Rawji et al., 2018). These cells become more immunogenic, produce inflammatory cytokines and reactive oxygen species, and thus, have a deleterious phenotype (Hammond et al., 2019). In the context of demyelination, aged microglial cells fail to take up myelin debris efficiently by phagocytosis (Ritzel et al., 2015; Rawji et al., 2018).

Interestingly, the process can be reversed, as old mice exposed to a youthful systemic environment *via* heterochronic parabiosis recover a remyelination potential similar to that of young mice (Ruckh et al., 2012). The clearance of myelin debris and remyelination can also be restored in old mice by systemic injections of niacin, which acts by upregulating CD36 (Rawji et al., 2020). Fasting and treatment with metformin (a fasting mimetic drug) also lead to a recovery of OPC responsiveness to differentiation signals in old mice (Neumann et al., 2019).

### Inflammation

CNS injury triggers a cascade of cellular and molecular events leading to inflammation. In MS, breakdown of the brain–blood barrier allows autoreactive T lymphocytes and macrophages to infiltrate the brain, increasing local levels of proinflammatory cytokines. Glial cells also make an active contribution to these environmental changes. Inflammation may itself cause demyelination, as in MS, in which leptomeningeal immune cell infiltration and compartmentalized inflammation within the subarachnoid space are tightly associated with the development of cortical lesions (Choi et al., 2012; Magliozzi et al., 2018).

**Figure 2:**
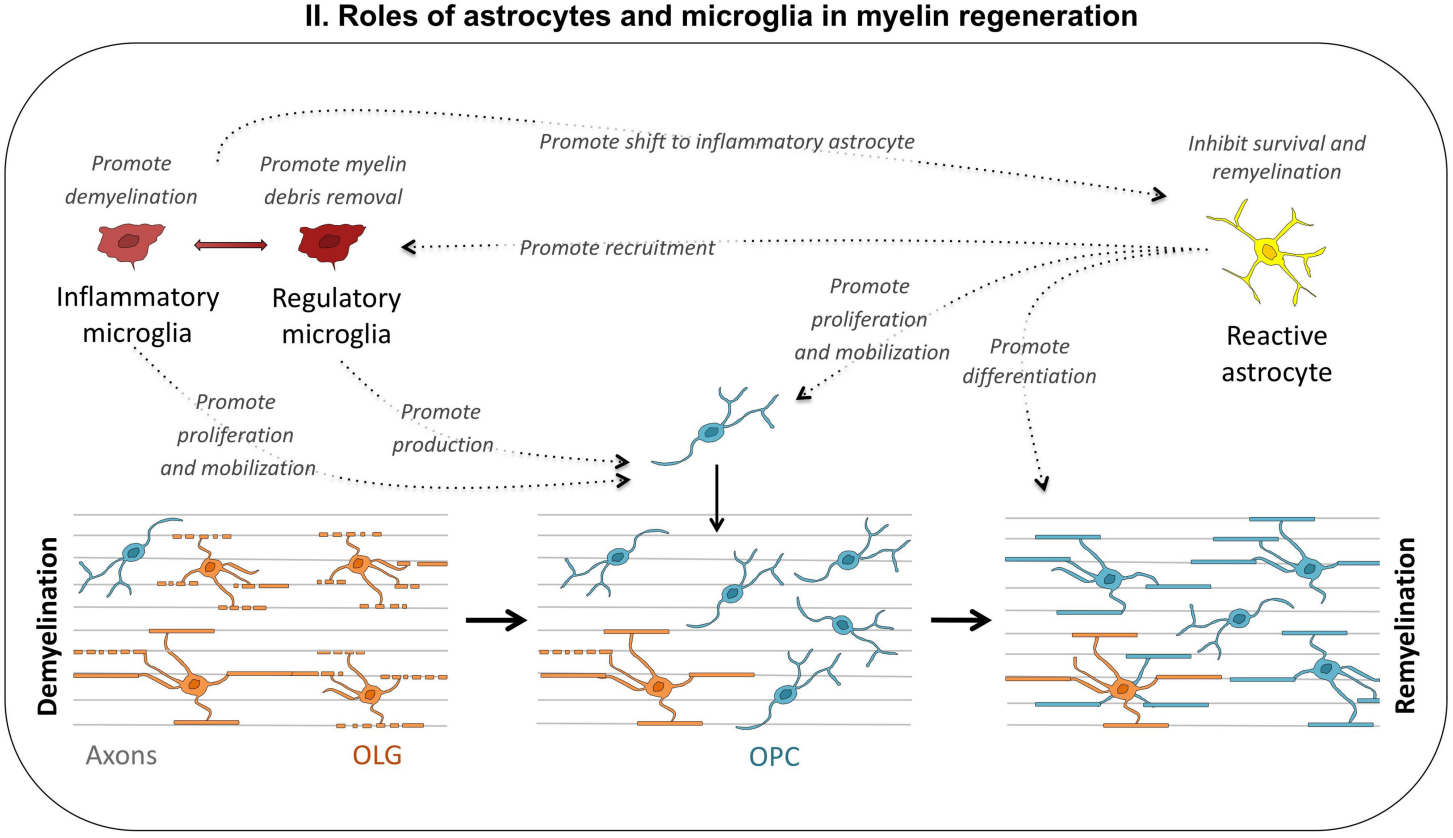
Roles of astrocytes and microglia in myelin regeneration. Depending on environmental cues, microglial cells may adopt different phenotypes, from proinflammatory to regulatory. Inflammatory microglia promotes demyelination and astrocyte reactivity, but may also be useful to the repair process, by stimulating OPC proliferation and mobilization. Regulatory microglia promotes remyelination by enhancing debris removal and OPC production. Reactive astrocytes also play multiple roles: they inhibit remyelination through inflammatory cytokine production and ECM modifications, promote myelin debris removal by recruiting microglia, and stimulate oligodendrogenesis.

Inflammatory mediators may have a negative or positive effect on progenitor cell-mediated remyelination. Acute inflammation is required for the correct remyelination of demyelinated lesions (Prineas et al., 1989; Foote and Blakemore, 2005; McMurran et al., 2016). In the cuprizone model, a lack of TNFα or MHC-2 leads to low levels of OPC proliferation and remyelination failure (Arnett et al., 2001, 2003). The delayed but prolonged expression of cytokines such as IL1β, Il6, and TNFα is associated with delayed remyelination in old rats (Zhao et al., 2006). Overall, these data suggest that early acute inflammation is required for the correct recruitment of OPCs to the lesion site, but that chronic inflammation may impede remyelination (Figure 2).

Inflammation can inhibit remyelination in several ways: (1) neural progenitors contributing to remyelination may be directly attacked by inflammatory cues or immune cells (Imitola et al., 2003, 2004). Activated T lymphocytes induce the progressive collapse of the process and the apoptotic death of neural progenitors and OPCs *via* the secretion of semaphorin (Giraudon et al., 2004); (2) inflammation affects axonal integrity, in turn altering axon–OPC communication during remyelination; and (3) the extensive remodeling of the extracellular matrix (ECM) associated with sustained inflammation renders the microenvironment nonpermissive for remyelination, mostly by preventing OLG differentiation. These inhibitory signals, secreted into the ECM, are generated principally by microglial cells and astrocytes activated upon injury. They have multiple complex functions in pathological conditions. Both microglia and astrocytes can present different phenotypes, behaving like Dr. Jekyll and Mr. Hyde (Figure 2).

#### Deleterious Effects of Astrocytes on Remyelination

A transcriptomic analysis of astrocytes activated by different types of insults (ischemia, LPS injection) revealed that two types of reactive astrocytes could be distinguished: A1 astrocytes with detrimental effects on cell survival and regeneration and A2 astrocytes presenting protective effects (Zamanian et al., 2012). Astrocyte reactivity upon demyelination is greater in white matter than in gray matter, probably due to the differential induction of various factors. Astrocyte reactivity is regulated by proinflammatory cytokines and myelin debris. Myelin debris are more abundant in white matter, contributing to higher levels of astrocyte activation. In addition, microglial reactivity occurs later and is weaker in the gray matter, resulting in lower levels of cytokine production (Gudi et al., 2009; Buschmann et al., 2012; Werkman et al., 2020). The activation state of astrocytes determines their permissive or inhibitory influence on remyelination.

Astrogliosis is one of the hallmarks of MS. Reactive astrocytes have been shown to increase the recruitment of OPCs to the lesion *via* the secretion of chemokines, such as CXCL1, 8, and 10 (Omari et al., 2005). They also promote the recruitment of microglial cells *via* CXCL10, thereby regulating myelin debris clearance (Skripuletz et al., 2013). In this way, astrocytes contribute to the repair process. Conversely, they also promote peripheral immune cell recruitment, thereby enhancing demyelination (Brambilla et al., 2014). Astrocytes can also regulate the myelin-specific autoreactive response of effector T cells *via* interleukin secretion (Correale and Farez, 2015). In EAE, astrocytes display impaired glutamate transporter expression, leading to deficiencies of glutamate uptake and excitotoxicity (Ohgoh et al., 2002).

Finally, astrocytes make a major contribution to deleterious ECM modifications through their production of hyaluronan, chondroitin sulfate proteoglycan (CSPG), fibronectin, and BMP, all of which inhibit OPC maturation and remyelination (Back et al., 2005; Pendleton et al., 2013; Harlow and Macklin, 2014). Fibronectin accumulates in demyelinated lesions and prevents OPC differentiation in the EAE model, but not in toxin-induced demyelination, suggesting that fibronectin aggregation is mediated by inflammation. In MS patients, fibronectin aggregates are found in chronic lesions but not in remyelinated lesions (Stoffels et al., 2013). Fibronectin may therefore contribute to remyelination failure.

In addition, these changes to ECM composition increase tissue stiffness, which has been shown to downregulate OPC proliferation and differentiation (Jagielska et al., 2012, 2017; Urbanski et al., 2016; Segel et al., 2019). Tissue stiffness is higher in chronic lesions than in actively remyelinating lesions in MS patients; similarly, tissue stiffness decreases slightly in the acute cuprizone model, but increases in the chronic model with long-term administration (Urbanski et al., 2019).

#### Roles of Microglia in Remyelination

Microglial cells are the resident immune cells of the CNS, and as such, they are the sensors of pathological events. They are, thus, among the first cells to be activated upon injury. Activated microglia proliferate and migrate to the lesion site, where they accumulate, with both beneficial and detrimental functions. Microglial ablation during the symptomatic phase of EAE has been shown to reduce CNS inflammation and promote recovery (Nissen et al., 2018), but impeding microglial function may also limit remyelination (Stangel et al., 2017; Lloyd and Miron, 2019). Indeed, microglia are crucial for the elimination of myelin debris by phagocytosis, a prerequisite for remyelination (Napoli and Neumann, 2010; Voß et al., 2012; Lampron et al., 2015).

Activated microglia adopt different phenotypes according to the combination of cytokines and factors they express. Traditionally, microglia have been categorized into M1 (proinflammatory) and M2 (immunomodulatory), although this classification now appears too simplistic. Indeed, the development of single-cell RNA sequencing analyses has led to the detection of microglial heterogeneity in both physiological and pathological conditions, suggesting that microglial activation is a dynamic response involving transcriptionally and spatially different subpopulations (Keren-Shaul et al., 2017; Hammond et al., 2019; Plemel et al., 2020). In the LPC demyelinating model, different phenotypes predominate at different stages of demyelination/remyelination, with an initial proinflammatory signature at the time of OPC recruitment, followed by a shift to a more regenerative state during remyelination (Miron et al., 2013). Inflammatory microglial necroptosis occurs, shutting down proinflammatory signals, and is followed by the repopulation of the lesion with regulatory microglia, a process required for efficient remyelination (Lloyd et al., 2019). Regulatory microglia can also promote SVZ-derived oligodendrogenesis and remyelination *via* Wnt7a production (Mecha et al., 2020).

Inflammatory microglia have several deleterious effects, including shifting astrocytes from a protective to an inflammatory phenotype *via* the secretion of extracellular vesicles and cytokines, such as IL1a, TNFα, and C1q. The astrocytes lose their ability to promote neuronal survival, become neurotoxic, block OLG differentiation, and enhance OLG death (Liddelow et al., 2017; Lombardi et al., 2019). Furthermore, like astrocytes, activated microglia secrete chemokines of the CXC and CC family, contributing to the intracerebral recruitment of T cells and antigen-presenting cells, such as macrophages and dendritic cells (O'Loughlin et al., 2018).

As mentioned above, microglia are sensitive to aging, becoming increasingly inflammatory and less effective at the phagocytosis of myelin debris, thereby contributing to remyelination failure.

## Remyelination in Humans: Observations From MS Patients

MS is an autoimmune disease leading to myelin destruction and neuronal dysfunction, finally triggering neurodegeneration. The pathological hallmark of MS is the occurrence of focal demyelinated lesions (or plaques) disseminated throughout the CNS, causing diverse functional deficits according to their location. These plaques can be classified into several categories on the basis of their inflammatory properties and the presence or absence of ongoing demyelination (Kuhlmann et al., 2017). Active lesions are characterized by the presence of macrophages/microglia throughout the lesion. In mixed active/inactive lesions, these immune cells are present only at the border of the lesion, and chronic lesions are hypocellular, with an almost complete absence of macrophages/microglia. The first two categories can be subdivided into demyelinated lesions (ongoing myelin destruction visible as myelin degradation products in the cytoplasm of macrophages/microglia) and postdemyelinating lesions.

### Evidence of Heterogeneous and Variable Myelin Repair in MS Patients

Observations of postmortem tissues from MS patients long ago raised the possibility of spontaneous myelin repair in humans (Prineas and Connell, 1979; Prineas et al., 1984). Remyelinated areas have thinner myelin sheaths, characterized by myelin of a paler color, and are known as “shadow plaques.” Lesions more frequently display partial remyelination, usually starting from the periphery; such partial remyelination is observed in all lesion types, but varies considerably between lesions and between patients (Patrikios et al., 2006; Patani et al., 2007). Extensive sampling of brain tissues from MS patients has suggested that remyelination may be more extensive than previously thought, with a mean of 47% remyelination in the white matter. Within the white matter, 22% of lesions were found to be shadow plaques, 73% were partially remyelinated, and only 5% were completely demyelinated (Patani et al., 2007).

Independently of chronicity and location, some MS patients are “good remyelinators” and others are “bad remyelinators” (Patrikios et al., 2006; Bodini et al., 2016). Little is known about the factors underlying this variability in myelin repair. The regenerative process is probably influenced by both genetic and environmental factors, although no specific genes or factors involved in this process have yet been clearly identified. Aging obviously plays a role in decreasing the efficiency of repair in chronic MS, probably through mechanisms reminiscent of those observed in animal models (see *Aging* section), but it cannot provide a complete explanation. Both disease duration and the location of the lesions affect remyelination potential. Many studies have suggested that remyelination is more prominent at the beginning of the disease than in chronic MS, in which the extent of remyelination is often limited (Prineas et al., 1993; Goldschmidt et al., 2009; Chen et al., 2013; Frischer et al., 2015). Depletion of the OPC pool through repeated demyelination is another possible explanation, although sustained demyelination rather than repeated insults is required to deplete OPCs and impair remyelination in rodent models (Penderis et al., 2003).

A recent study suggested that there may be multiple reasons for remyelination failure in MS patients, according to lesion stage: in a subset of active lesions, a lack of myelin sheath formation was observed despite the presence of mature OLGs, whereas in inactive lesions, OLG loss and a hostile microenvironment were identified as the major causes of remyelination failure (Heß et al., 2020).

Subcortical lesions display more extensive remyelination than periventricular and cerebellar lesions, which are often poorly remyelinated (Goldschmidt et al., 2009). Lesions in cortical areas are consistently more extensively remyelinated than white matter lesions (Albert et al., 2007; Strijbis et al., 2017), as clearly shown by the analysis of leukocortical regions, which embrace both white and gray matter (Chang et al., 2012). Cortical lesions are observed in all forms of MS, but are most prominent in long-term progressive MS and are more strongly associated with functional disability than white matter lesions. There is evidence to suggest that environmental cues, produced by astrocytes and microglia in particular, may underlie these differences in remyelination potential (Chang et al., 2012). Indeed, cortical lesions often lack the pathological hallmarks of MS white matter lesions, instead displaying only low levels of inflammation and gliosis (Peterson et al., 2001; Bø et al., 2003).

Finally, the sex of the patient may affect disease susceptibility, progression, and regeneration. Indeed, MS affects three times more women than men, but appears to follow a less aggressive course in women (Confavreux and Vukusic, 2006a). The rate of relapses decreases strongly during the last trimester of pregnancy, when estrogen and progesterone levels are at their highest, whereas there is a rebound after delivery, coinciding with the decrease in hormone levels (Confavreux et al., 1998). *In vitro* experiments and preclinical studies also support the hypothesis that sex hormones may improve remyelination (for a review, see El-Etr et al., 2011).

It therefore appears likely that several mechanisms and factors contribute to impaired remyelination, ranging from genetic background to neuropathological subtype.

### Cell Sources for Myelin Repair in MS Patients

The identification of cells contributing to myelin repair is of course more difficult and less reliable in humans than in animal models. However, a number of observations suggest that several cell types may be involved. NG2-positive OPCs are present in MS lesions and can produce new OLGs characterized by short internodes (Chang et al., 2002, 2012). However, in chronic white matter lesions, OPC differentiation into OLGs like that observed in early active lesions does not occur (Kuhlmann et al., 2008), suggesting that inhibitory factors block differentiation. In the spinal cord of MS patients, myelin sheaths labeled with P0, a major constituent of peripheral nervous system myelin, can be detected, indicating the contribution of Schwann cells to CNS remyelination (Itoyama et al., 1983, 1985). Finally, SVZ activation is observed in MS patients, with increases in oligodendrogenesis and the ectopic migration of progenitors that are thought to be involved in remyelination (Nait-Oumesmar et al., 2007; Wu et al., 2009). Based on these observations, it seems likely that myelin repair in the human brain can, as in rodents, proceed from different cell sources, including OPCs, Schwann cells, and SVZ-derived progenitors.

Recent studies using ^14^C levels to date the birth of cells concluded that, in healthy adult human white matter, only a very small number of new OLGs are generated (Yeung et al., 2019). More surprisingly, newborn OLGs were almost undetectable in the remyelinated lesions of MS patients, except in very aggressive forms of the disease (Yeung et al., 2019). The absence of new OLGs in shadow plaques led the authors to conclude that myelin repair in the human brain does not stem from OPCs, instead originating from surviving OLGs that form new myelin sheaths. This contrasts sharply with observations and demonstrations in animal models, in which remyelination from mature OLGs is restricted to very particular conditions (Duncan et al., 2018; Bacmeister et al., 2020). This finding also calls into question the use of rodent models in the design of therapeutic strategies and suggests that the use of larger animal models, such as cats and nonhuman primates, should be considered (Duncan et al., 2018). Indeed, no single animal model can recapitulate the entire spectrum of heterogeneity for human MS lesions and repair mechanisms, and the best option would be to use several models. A role for mature OLGs in remyelination would have important consequences for the design of new therapeutic strategies: in this case, efforts should be made to promote OLG survival and plasticity.

However, many questions remain unresolved due to several obstacles, making it impossible to draw definitive conclusions from these studies. First, it is difficult to date the beginning of the disease and lesion initiation in humans. Second, if histology is not coupled with longitudinal *in vivo* imaging, it is not possible to conclude with certainty that remyelination has occurred. The histological hallmark of remyelination is the observation of shadow plaques, visible because the regenerated myelin sheath is thinner than the original one; however, this assumption is currently questioned (Neumann et al., 2020). In particular, in areas in which the axon diameter is small, as in the CC, the difference is minimal, and remyelinated areas may be very similar to healthy areas, leading to an underestimation of remyelination rates. Conversely, a shadow plaque may not necessarily be associated with remyelination: paler coloration may result from partial demyelination or a decrease in axonal density. It is not, therefore, yet possible to rule out a contribution of OPCs to myelin repair in the human brain.

## Post-lesional Plasticity and Myelin Repair: Translation Into Therapy

MS is primarily an immune-mediated disease, and until very recently, treatments were designed to target immune cells and inflammation. These immunomodulation therapies proved efficient for limiting and reducing lesion formation and relapse rate but failed to prevent the progression of the disease toward neuron loss and irreversible disability.

As mentioned in the *Remyelination Is a Major Issue for Preventing Neurodegeneration and Irreversible Losses of Function* section, many studies have provided support for the idea that targeting remyelination is a sound strategy for promoting functional recovery. Here, we focus on strategies promoting myelin repair from endogenous cell sources; we do not, therefore, consider cell transplantation approaches. Bearing in mind the various factors shown to be involved in the production of new OLGs and in remyelination failure, several strategies for enhancing spontaneous repair may be considered.

### Promoting OPC Recruitment and/or Myelin Formation

Boosting OPC proliferation and differentiation appears to be a straightforward strategy for promoting remyelination. However, the reality is more complex. Lesions at different stages coexist in MS patients, and neuropathological studies indicate that there is a high degree of heterogeneity between lesions (Lucchinetti et al., 1999, 2000), with some lacking OPCs (Boyd et al., 2013) and others full of OPCs with blocked differentiation programs (Wolswijk, 1998; Kuhlmann et al., 2008). It is therefore difficult to determine the most appropriate time window for the use of proliferating or differentiating agents. Indeed, promoting OPC proliferation (potentially inhibiting cell differentiation) would be at best useless and possibly even detrimental for lesions containing progenitors unable to differentiate into myelinating OLGs; conversely, promoting OLG differentiation in lesions with only a few rare OPCs would be highly inefficient.

Two approaches currently exist for the development of new treatments targeting OPCs and OLGs (Table 1). The first is based on our current knowledge of factors or receptors governing oligodendrogenesis and myelination. The thyroid hormone and retinoic X receptor gamma (RXRγ) have excited considerable interest in this context, due to their promyelinating effects (Harsan et al., 2008; Huang et al., 2011; de la Fuente et al., 2015). The second approach is blind to mechanisms of action and based on the *in vitro* screening of compounds from libraries. This approach was made possible by the recent development of high-throughput platforms assessing myelination (Mei et al., 2014; Lariosa-Willingham and Leonoudakis, 2018). Several promising small molecules have been identified, including miconazole, clobetazole, clemastine, and benzatropine, highlighting new pathways regulating OLG differentiation (the MAP kinase, glucocorticoid receptor, and muscarinic acetylcholine receptor pathways) (Deshmukh et al., 2013; Najm et al., 2015).

**Table 1:** Compounds and therapies promoting remyelination.

			**Preclinical studies**		**Clinical trial**		
	**Compounds**/ **therapy**	**Mode of action**	**Experimental models**	**Effects observed**	**Trial**	**Results**	**References**
**Pro-myelinating compounds**	Retinoic acid, bexarotene	RXR agonist; promotes the development of regulatory Tcells and suppresses the development of T helper 17 cells.	Ethidium bromide in rats; LPC in mice	Promote OPC differentiation and remyelination	Phase 2 “CCMR one”	Slightly improved lesion remyelination (MRI), reduced visual evoked potential latency but side effects (hypothyroidism, hypertriglyceridemia)	Huang et al., 2011; Chandraratna et al., 2016
	Thyroïd hormone, Sobetirome	Thyroïd hormone agonist	Cuprizone in mice; EAE in mice	Protects against demyelination and axonal degeneration, improves remyelination and clinical outcome.	Phase 1	Short-term safety	Harsan et al., 2008; Wooliscroft et al., 2020; Chaudhary et al., 2021
	Clemastin	H1 receptor but act as anti-M1 mAchR	LPC in mice, Cuprizone in mice	Promotes OPC differentiation and remyelination	Phase 2 “ReBUILD” and “ReCOVER”	Slightly reduced evoked potential latency but no clinical improvement	Mei et al., 2014; Li et al., 2015; Green et al., 2017
	Benzatropin	Anticholinergic (M1/M3 receptor antagonist)	Cuprizone and EAE in mice	Enhances remyelination and decreases disease severity	No		Deshmukh et al., 2013
	Miconazole	Antifungal drug acting *via* MAPK and *via* cholesterol biosynthesis	LPC and EAE in mice	Promotes OPC differentiation and remyelination	No		Najm et al., 2015
	Clobetazol	Immunosuppressor, acts *via* glucocorticoid receptor signaling	LPC and EAE in mice	Promotes OPC differentiation and remyelination; immunosuppression	No		Najm et al., 2015
	Bazedoxifene	Selective estrogen receptor modulator, but acts *via* cholesterol biosynthesis	LPC in mice	Promotes OPC differentiation and remyelination	Phase2	Ongoing	Rankin et al., 2019
	Olesoxime	Mitochondria, microtubule	Cuprisone and LPC in mice	Promotes OPC differentiation and accelerates remyelination	Phase 1		Magalon et al., 2012, 2016
	Biotin	Co-factor for enzymze involved in fatty acid synthesis and energy production.	Rat OPC primary culture; biotidinase KO mice	Promotes myelin synthesis and protects against axonal degeneration	Phase 3	Failed to improve disability in patients with progressive MS	Pindolia et al., 2012; Sedel et al., 2016; Tourbah et al., 2016; Cree et al., 2020; Cui et al., 2020
	Neuronal activity	Local translation of MBP	LPC in mice	Promotes OPC differentiation and functional improvement	Transorbital electrical stimulation	Ongoing	Ortiz et al., 2019
**Suppression of inhibitory signals**	Temelimab	Monoclonal antibody GNbAC1 against the envelop of human endogenous retrovirus prevents TLR4 activation	Human primary OPC culture	Env-mediated stimulation of TLR4 on OPC induces inflammatory cytokines and prevent myelin protein expression	Phase 2	Decreased cortical atrophy, slight effect on remyelination	Kremer et al., 2013; Derfuss et al., 2015
	Opicinumab	Anti-LINGO-1, inhibits RhoA activation	EAE in rats and mice	Increased axonal integrity and remyelination, improved clinical score	phase 2 “Affinity” “Synergy” and “Renew”	Reduced evoked potential latency in acute optic neuritis but failure to improve physical and cognitive function in RRMS patients	Mi et al., 2007; Klistorner et al., 2018; Cadavid et al., 2019
	Metformin	Anti-diabetic, rejuvenating	Ethidium bromide in rats	Reverses age-related changes in OPCs, improves remyelination in aged animals	Phase 1	Ongoing	Neumann et al., 2019
**Suppression of inhibitory signals**	DHA and EPA	Polyunsaturated fatty acids. Switch microglia phenotype	Cuprizone, culture	Enhance myelin debris phagocytosis, reduce demyelination, improve cognitive function			Chen et al., 2014
	Endocannabinoid 2-AG	Activates CB1, CB2, and TRVP1 receptors	TMEV-IDD viral murine model	Enhances the clearance of myelin debris, promotes OPC differentiation			Mecha et al., 2019
	Niacin (vitamin B3)	Regulates CD36 expression	LPC, culture	Increases myelin debris phagocytosis by macrophages and microglia and improves remyelination			Rawji et al., 2020
	rHIgM22	IgM antibody binds CNS myelin	TMEV-IDD viral murine model, cuprizone, culture	Stimulates myelin debris phagocytosis by microglial cells, promotes remyelination	Phase 1	Well tolerated, positive trend on clinically stable MS patients	Warrington et al., 2007; Eisen et al., 2017; Mullin et al., 2017; Zorina et al., 2018
	Dietary restriction	Anti-inflammatory, rejuvenating	EAE and cuprizone in mice	Reduces pro-inflammatory cytokines, promotes OPC regeneration and remyelination, reduces clinical severity	Special diets	Ongoing	Choi et al., 2016; Neumann et al., 2019

Bazedoxifene (BZA), a selective estrogen receptor modulator, has been identified as a promyelinating agent, with further studies validating its remyelinating effect *in vivo* after demyelinating insults (Rankin et al., 2019). This molecule is now being evaluated in a phase 2 clinical trial on MS patients. One recent study revealed a common mechanism of action for these diverse compounds, independent of their canonical pathways: they all interfere with the cholesterol biosynthesis pathway, leading to the accumulation of 8,9-unsaturated sterols, which stimulate the differentiation of OPCs into myelinating OLGs (Hubler et al., 2018; Rankin et al., 2019).

A small cholesterol-like compound, olesoxime, has been shown to promote oligodendrocyte maturation, remyelination, and functional recovery in rodent models of demyelination, *via* binding to mitochondria and the modulation of ROS levels (Magalon et al., 2012, 2016). Great hopes were raised by biotin, an essential cofactor for fatty-acid synthesis and energy production that was found to have beneficial effects against progressive MS in a pilot study (Sedel et al., 2016; Tourbah et al., 2016); unfortunately, it provided no improvement in clinical outcome in a phase 3 clinical trial.

Recent studies in animal models have highlighted the crucial role of neuronal activity in OPC proliferation and *de novo* myelination (for a review, see Sampaio-Baptista and Johansen-Berg, 2017). Based on these observations, two clinical trials were launched on electrical stimulation of the optic nerve in patients suffering from acute optic neuritis.

Finally, given the minimal *de novo* generation of OLGs from OPCs in the brains of adult humans, including MS patients (Yeung et al., 2014, 2019), efforts should also be made to identify factors capable of boosting mature OLG survival and plasticity during acute demyelination. Creatine was found to be effective for this purpose in the lysolecithin model, in which it protected OLGs against caspase-dependent apoptosis by enhancing mitochondrial function (Chamberlain et al., 2017).

If OLGs are found to play a major role in remyelination in the human brain, they should, indeed, be considered as new targets for drug development.

### Removing Inhibitory Signals and Reversing the Effects of Aging

Blocking or removing signals that inhibit OLG differentiation and myelination should render the microenvironment more permissive for regeneration.

The Notch, Wnt, and LINGO-1 signaling pathways have been identified as major inhibitors of CNS remyelination (Wang et al., 1998; Mi et al., 2005; Zhang et al., 2009). Preclinical studies provided strong evidence that LINGO-1 inhibition enhances repair in demyelinating disease (Bourikas et al., 2010; Mi et al., 2013; Sun et al., 2015). Clinical trials with LINGO-1 antagonists have provided encouraging preliminary results, at least for optic neuritis.

The CSPG and hyaluronan produced by reactive astrocytes exert potent inhibitory signals on OLG development (Back et al., 2005; Lau et al., 2012, 2013; Pu et al., 2018). Most drugs known to induce OPC differentiation *in vitro* are ineffective in the presence of CSPG (Keough et al., 2016). MS lesions are enriched in CSPG, indicating that they may be good targets for neutralization to improve remyelination. In preclinical studies, CSPG biosynthesis inhibitors were shown to be efficient for rescuing OPC differentiation *in vitro* and accelerating remyelination in mice (Lau et al., 2012; Keough et al., 2016). A better knowledge of CSPG and the signaling cascades operating in OPCs will be required for the design of future therapeutic strategies. Similarly, low molecular weight hyaluronans impair OLG differentiation and remyelination, and their concentrations are high in chronic MS lesions (Back et al., 2005; Sloane et al., 2010). The inhibition of low molecular weight hyaluronans may, therefore, also be therapeutically relevant. Finally, the recent discovery of OPC mechanosensitivity and of the impact of tissue stiffness on OLG maturation and myelination opens up new perspectives for treatment (Makhija et al., 2020), although we will need to understand much more about the mechanobiology of OPCs before translation into clinical practice.

Myelin debris is a potent inhibitor of remyelination. The transmembrane protein EphrinB3 has been shown to be an important mediator of this inhibition of OLG maturation; the masking of EphrinB3 epitopes promotes remyelination in a focal demyelination rat model (Syed et al., 2016). This discovery has been patented and may lead to the future development of new therapeutic neutralizing antibodies. Improving myelin clearance is a major challenge. No drug is currently available for specifically preventing the aging-induced decrease in microglial phagocytosis, although several candidate molecules have been identified (Pinto and Fernandes, 2020). These molecules include two polyunsaturated fatty acids (DHA and EPA) (Chen et al., 2014), the endocannabinoid 2-AG (Mecha et al., 2019), and an experimental recombinant human antibody rhIGM22 (Zorina et al., 2018). Vitamin B_3_ (niacin) also seems to be a promising compound for safely enhancing myelin phagocytosis by microglia (Rawji et al., 2020). TREM2 is another potentially interesting candidate target. It is expressed by microglia and is involved in the proliferation and phagocytic activity of these cells. One very recent study of a TREM2 agonistic antibody in the cuprizone model reported enhanced myelin debris uptake and degradation, together with improved remyelination (Cignarella et al., 2020). However, as microglia also take up synapses by phagocytosis, a fine balance must be achieved to promote the phagocytosis of myelin debris specifically, without eliminating synapses.

Another approach could involve targeting lipid metabolism, as the uptake of too much cholesterol-rich myelin debris converts microglia into cells with a proinflammatory profile. Molecules triggering the upregulation of ATP-binding cassette (ABC) transporters promote lipid efflux from human macrophages and are, thus, potentially good candidates (Pinto and Fernandes, 2020).

Finally, approaches fighting cellular aging may represent an interesting strategy. Preclinical studies have shown that OPCs can be rejuvenated by alternate-day fasting or by treatment with the fasting mimetic metformin (Neumann et al., 2019). Interestingly, in EAE mice, a fasting mimicking diet strongly reduces clinical severity and inflammation and promotes axonal remyelination (Choi et al., 2016). Several clinical trials targeting dietary interventions have been completed, but they provided insufficient evidence of efficacy for translation into clinical practice. However, most of these trials included limited numbers of patients and short treatment durations (Evans et al., 2019). Other clinical trials are still underway, testing various specific regimens, such as a ketogenic diet and intermittent fasting. These approaches, with few deleterious side effects, may be an interesting complement to drug administration.

## Conclusion

In recent years, the discovery of several compounds that effectively promote remyelination and provide neuroprotection in animal models has raised hopes for the development of new treatments for MS patients, particularly for preventing or treating the progressive form of the disease, for which very few options are currently available. However, many obstacles will need to be overcome before this goal can be achieved.

First, it should be remembered that no animal model fully reproduces all the characteristics of MS in humans, and young rodents are used, at ages at which regenerative potential is optimal. Drugs found to be effective in such experimental designs may be less effective in less favorable conditions. The use of larger animal models in preclinical studies may be required to overcome this problem.

It is reasonable to assume that the combination of immunomodulatory treatments with compounds alleviating inhibitory signals for remyelination, together with the use of other treatments stimulating OLG differentiation, would have the greatest effect. However, the design of such combinatorial therapies is complicated and they are difficult to test in clinical trials. For instance, it is not immediately obvious whether the various drugs should be administered simultaneously or in a particular time window. The negative effects of permanently stimulating OPC differentiation should also be taken into account, because such treatment may ultimately lead to the depletion of the OPC pool. Will all patients benefit from the same protocol, or do we need to characterize the clinical and neuropathological specificities of each patient more precisely to propose adapted therapy? If we are to achieve this ambitious objective, we will first need to identify valuable biological markers and to develop imaging techniques of better predictive value. Indeed, demyelination/remyelination follow-up in patients included in clinical trials requires improvement, to ensure that treatment efficacy is properly evaluated. MRI techniques are still being developed, to improve signal specificity, but reliable blood biomarkers are still lacking, and neurophysiological measurements will also be required, to estimate functional recovery.

## Author Contributions

MC wrote the original draft and MF generated the figures. All authors contributed to the writing of this article, approved the submitted version, involved in developing the plan for the article, and in reviewing and editing the manuscript.

## Conflict of Interest

The authors declare that the research was conducted in the absence of any commercial or financial relationships that could be construed as a potential conflict of interest.
